# Radiomic model based on magnetic resonance imaging for predicting pathological complete response after neoadjuvant chemotherapy in breast cancer patients

**DOI:** 10.3389/fonc.2023.1249339

**Published:** 2024-01-31

**Authors:** Yimiao Yu, Zhibo Wang, Qi Wang, Xiaohui Su, Zhenghao Li, Ruifeng Wang, Tianhui Guo, Wen Gao, Haiji Wang, Biyuan Zhang

**Affiliations:** ^1^ Department of Radiation Oncology, The Affiliated Hospital of Qingdao University, Qingdao, China; ^2^ Department of Gastroenterological Surgery, The Affiliated Hospital of Qingdao University, Qingdao, China; ^3^ Department of Galactophore, The Affiliated Hospital of Qingdao University, Qingdao, China

**Keywords:** breast cancer, radiomics, MRI, neoadjuvant chemotherapy, pathological complete response

## Abstract

**Purpose:**

To establish a model combining radiomic and clinicopathological factors based on magnetic resonance imaging to predict pathological complete response (pCR) after neoadjuvant chemotherapy in breast cancer patients.

**Method:**

MRI images and clinicopathologic data of 329 eligible breast cancer patients from the Affiliated Hospital of Qingdao University from August 2018 to August 2022 were included in this study. All patients received neoadjuvant chemotherapy (NAC), and imaging examinations were performed before and after NAC. A total of 329 patients were randomly allocated to a training set and a test set at a ratio of 7:3. We mainly studied the following three types of prediction models: radiomic models, clinical models, and clinical-radiomic models. All models were evaluated using subject operating characteristic curve analysis and area under the curve (AUC), decision curve analysis (DCA) and calibration curves.

**Results:**

The AUCs of the clinical prediction model, independent imaging model and clinical combined imaging model in the training set were 0.864 0.968 and 0.984, and those in the test set were 0.724, 0.754 and 0.877, respectively. According to DCA and calibration curves, the clinical-radiomic model showed good predictive performance in both the training set and the test set, and we found that we had developed a more concise clinical-radiomic nomogram.

**Conclusion:**

We have developed a clinical-radiomic model by integrating radiomic features and clinical factors to predict pCR after NAC in breast cancer patients, thereby contributing to the personalized treatment of patients.

## 1 Introduction

According to GLOBOCAN 2020 database statistics, there were nearly 2.3 million new breast cancer cases and 685,000 breast cancer deaths in 2020, making breast cancer the most common cancer in women (1). Neoadjuvant chemotherapy may reduce the stage, improve the chance of surgery or improve the prognosis (2). The ideal state of neoadjuvant chemotherapy is to have no residual invasive tumor cells in the breast tissue resected after NAC or to achieve pCR. The realization of pCR is a strong proxy for recurrence and long-term survival risk (3), but there is currently no standard way to predict pCR. However, prospective clinical trials have demonstrated that the effectiveness and accuracy of SLNB after NAC is lower than expected in patients newly diagnosed with positive lymph nodes, and the false-negative rate is also higher. If noninvasive methods can be used to predict pCR, surgical trauma to patients can be avoided. To better predict the response of patients to NAC, mammography ultrasound, magnetic resonance and PET-CT are widely used. Due to the high resolution of magnetic resonance imaging, it is an imaging method with high specificity and sensitivity, so it is widely used (4). MRI is particularly sensitive to the diagnosis of breast cancer and the evaluation of its size, especially for young women less than 50 years of age, with a sensitivity of nearly 90%. At the same time, MRI is considerably better than ultrasound and mammography in the diagnosis of multicenter or multifocal breast cancer (5, 6). MRI images of breast cancer often include the axillary region, allowing simultaneous detection and diagnosis of axillary lymphadenopathy, especially in the evaluation of axillary zone III and internal milk lymphadenopathy; the approach is less dependent on the operator’s level of experience (5, 7).

In recent years, radiomics has been on the rise. By extracting multiple quantitative features from single or multiple medical imaging modes, radiomics can substantially enhance the discrimination and prediction potential of medical imaging by highlighting features invisible to the naked eye (8). The process of radiomics consists of several successive steps, and the methods used in each step determine the quality of the final model, so different methods may result in heterogeneous results that are difficult to compare (9). Radiomics research in the field of clinical diagnosis, treatment, and prognosis of cancer is encouraging; it includes immune response in patients with non-small cell lung cancer (10), microvascular infiltration in hepatocellular carcinoma (11), and nonrecurrent survival in patients with esophageal gastric cancer (12). In the field of breast cancer, radiomics has been widely used in the differentiation, prognosis, and recurrence risk of benign and malignant breast cancer (13–15). Several studies have demonstrated that radiomics models based on MRI have a good predictive effect on the prognosis of breast cancer patients (16–18). Previous studies have shown that MRI-based imaging models show good predictive performance, but the results are quite different. Moreover, this kind of research is still at a relatively early stage, so there are methodological and technical variations in the process of extracting image omics features.

MRI is currently common in neoadjuvant chemotherapy for breast cancer patients. Radiomic and deep learning are developing rapidly (19). There is little research on whether the combination of radiomics and clinicopathological features offers a better prognostic effect regarding breast cancer than radiomics alone or clinical models. In addition, few studies have used the combination of pre- and post-NAC images to predict pCR, and the number of patients included is small, so we will combine the above radiomic and clinical conditions in this study to construct a model to predict whether breast cancer patients achieve pCR after neoadjuvant therapy.

## 2 Materials and methods

This single-center, retrospective study was approved by our Institutional Review Board, and the requirement for written informed consent was waived.

### 2.1 Patient screening and study design

A total of 329 patients with breast cancer admitted to the Affiliated Hospital of Qingdao University from August 2018 to August 2022 were included in this retrospective study according to the following inclusion and exclusion criteria. The inclusion criteria were as follows: (a) breast cancer confirmed by puncture pathology; (b) DCE-MRI examination before and after neoadjuvant chemotherapy; (c) available clinical and pathological data; and (d) at least 4 cycles of neoadjuvant chemotherapy prior to radical surgery. The exclusion criteria were as follows: (a) loss of DCE-MRI images; (b) patients receiving neoadjuvant chemotherapy in other institutions; and (c) patients with other cancers during the same period.

A total of 329 patients were enrolled and assigned to two datasets (230 patients in the training and 99 patients in the validation) using computer-generated random numbers in a ratio of 7:3. According to the NCCN and CSCO guidelines, the neoadjuvant chemotherapy regimens for Her-2 positive patients include TCbHP (docetaxel, carboplatin, trastuzumab and pertuzumab), THP (docetaxel, trastuzumab and pertuzumab), TCbH (docetaxel, carboplatin and trastuzuma), and AC-THP (epirubicin, cyclophosphamide – paclitaxel, trastuzumab and pertuzumab); the regimens for triple-negative breast cancer include TAC (docetaxel, doxorubicin and cyclophosphamide), AT (epirubicin and docetaxel), TP (albumin paclitaxel and platinum), AC-T (epirubicin, cyclophosphamide – paclitaxel), and AC-TP (epirubicin, cyclophosphamide – paclitaxel, platinum); and the regimens for hormone receptor-positive patients include TAC, AT, and AC-T. By immunohistochemistry (IHC) of US-guided core biopsies, the tumor type and receptor status were confirmed. The patients underwent surgery within 6 weeks after the completion of NAC.

### 2.2 Clinical prediction model

Baseline clinical data including age, clinical T stage and N stage, menopausal state, estrogen receptor, progesterone receptor, Her-2, and Ki-67 were obtained from the hospital medical record system. Histopathological findings were assessed jointly by two pathologists with at least 10 years of diagnosis of breast disease. Age groups were classified as 40 years or older and 40 years or younger. In accordance with the eighth TNM staging criteria, T: Tumor indicates the extent of the primary tumor. N: Lymph node, represents the existence and extent of regional lymph node metastasis. Menopause in women refers to the permanent absence of menstruation caused by the cessation of ovarian function. Generally, menopause can be determined after 12 months. ER refers to estrogen receptor, PR refers to progesterone receptor, and the positive threshold of ER and PR immunohistochemical detection is ≥1%. Human epidermal growth factor receptor 2 (Her-2) positivity (+) is determined by the American Society of Clinical Oncology (ASCO) Guidelines. Ki-67 is a proliferative cell-associated nuclear antigen whose function is closely related to cell mitosis, and its classification is according to the proportion of positive cells. To evaluate the patient’s pathological response, we selected the Residual Cancer Burden (RCB) system recommended by the International Breast Collaboration Group, and RCB grade 0 represents PCR. Patients in the training group were classified into the pCR group (n=57) and the nonpCR group (n=174), and those in the test group were classified into the pCR group (n=24) and the nonpCR group (n=75). Differences in important variables between the training and test datasets were not statistically significant. We used the eight clinical features collected above to construct the clinical model.

### 2.3 Radiomics prediction model

#### 2.3.1 MRI acquisition

All the MRI examinations were performed with 1.5- or 3.0- Tesla scanners within two weeks before initiation of NAC and after completing NAC. A 3.0T double gradient superconducting MRI with a bilateral breast array coil (GE Health Care) was used. The patient had bilateral mammary glands naturally overhanging in the coil hole in the prone position, and relevant parameters were set. The sagittal position, transverse position and coronal position were routinely scanned, and multiphase enhanced breast volume imaging was used in the dynamic enhancement scan. A total of 8 phase scans were performed. Finally, high-resolution plain scan and dynamic contrast-enhanced imaging were obtained.

### 2.4 Radiomics feature extraction and model construction

**Figure 1:**
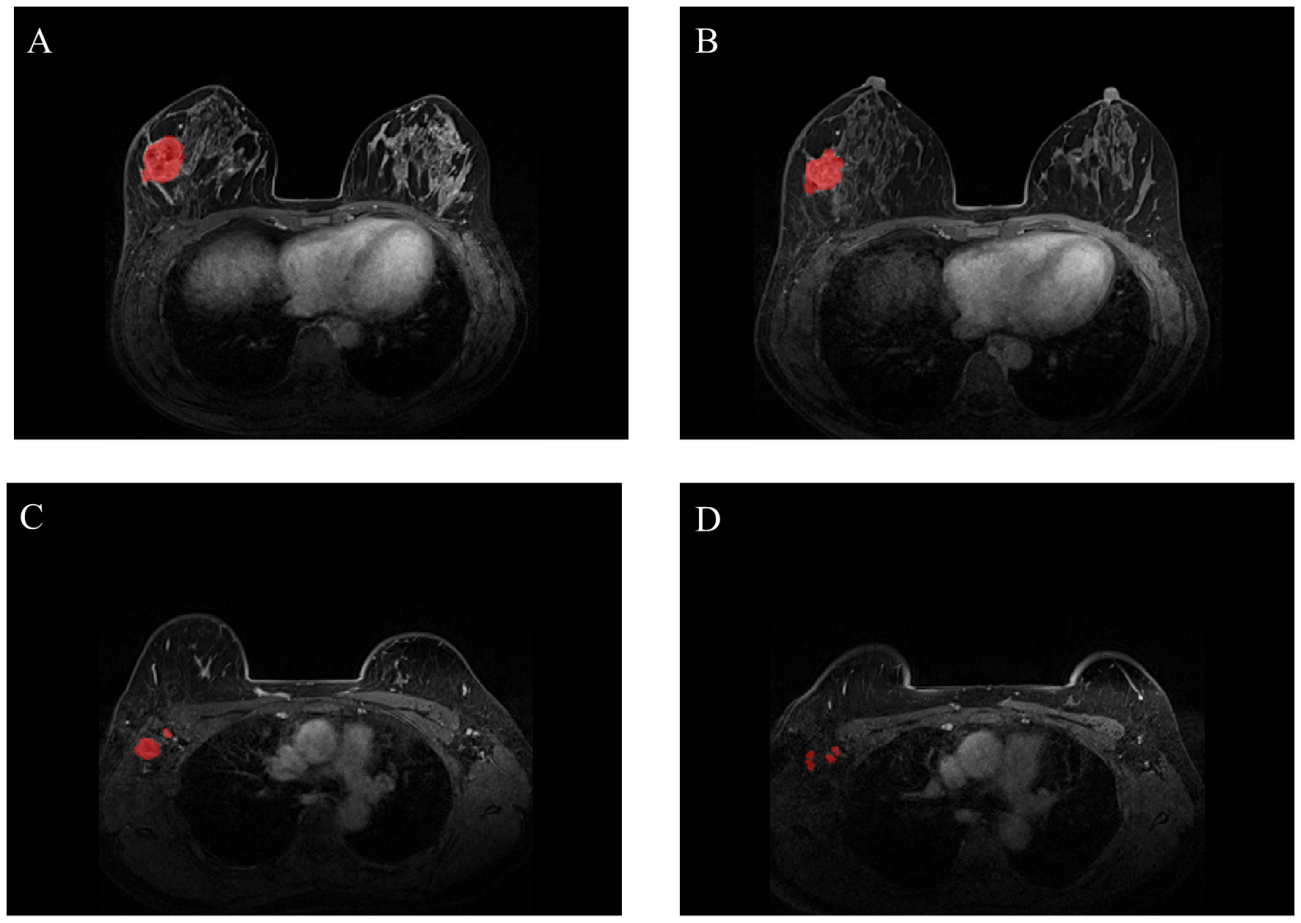
Representative images of breast tumors [**(A)** for pre-NAC; **(B)** for post-NAC] and representative images of axillary lymph nodes [**(C)** for pre-NAC; **(D)** for post-NAC]. The breast tumors and axillary lymph nodes were manually segmented (NAC: neoadjuvant chemotherapy).

Most breast cancers are shown most clearly in enhanced T1WI sequences, so the region of interest (ROI) (**Figure 1**) is only outlined in enhanced T1WI sequences to more accurately determine tumor boundaries and axillary lymph node distribution. To extract imaging features, two ROIs were selected, one for primary breast tumors and the other for axillary lymph nodes. We manually delineated ROI stratification in MRI using ITK-SNAP to delineate axillary lymph nodes and primary tumors before and after NAC treatment. ROI was sketched at all visible levels of the primary tumor and lymph nodes. Two doctors with at least 15 years of experience in reading breast MRI images were selected for ROI profiling, and any differences were resolved by consensus. If no tumor was visible on the post-NAC MRI, we performed manual segmentation based on the tumor bed and/or anatomic markers on the pre-NAC MRI. We used the Python software package PyRadiomics to extract the image radiomic data from the original image, including shape features, first-order features, and texture features, and then used the filter to calculate the high-order features according to the above features. We sketched the primary tumor and lymph nodes separately, then extracted the features separately, and finally analyzed all the features together. After feature extraction, Pearson’s correlation coefficient was used to select features, and then lasso regression was used for screening. Finally, according to the selected features, we used the scikit-learn package in Python 3.70 for model construction and evaluation, the training set for model construction and repeated cross-validation, and the test set for the final evaluation of the model. We chose logistic regression (LR), support vector machine (SVM), random forest (RF) and eXtreme Gradient Boosting (XGBoost) to develop the prediction model. The model established by pre-NAC MRI was R1. Similarly, we conducted the same modeling processing for post-NAC MRI, and the model was R2. We combined the advantages of R1 and R2 to build a new model.

### 2.5 Construction of the clinical-radiomic model

We combined predictors from the clinical model with optimal radiomic features from R1 and R2 to create a clinical-radiomic model, which was evaluated in a test set.

### 2.6 Statistical analysis

All statistical tests were conducted in Python 3.6 and SPSS 26. Independent t tests were used for continuous variables, and chi-square tests were used for categorical variables. The LASSO regression model was analyzed through the “Glmnet” package, and then the receiver operating characteristic (ROC) curve, decision curve (DCA) and calibration curve were drawn using the “Proc,” “Dca. R” and “rms” software packages, respectively. We evaluated the predictive performance of the model by analyzing receiver operating characteristic (ROC) curves and calculating the area under the curve (AUC). It is a performance index to measure the merits and demerits of the learner. The decision curve is intended to describe the entire forecast model or a test to see the net benefit of an intervention based on the model results, while the calibration curve is intended to avoid overfitting the model. For all analyses, P< 0. 05 was considered statistically significant, and all tests were two-sided.

## 3 Results

### 3.1 Characteristics of the enrolled patients

**Figure 2:**
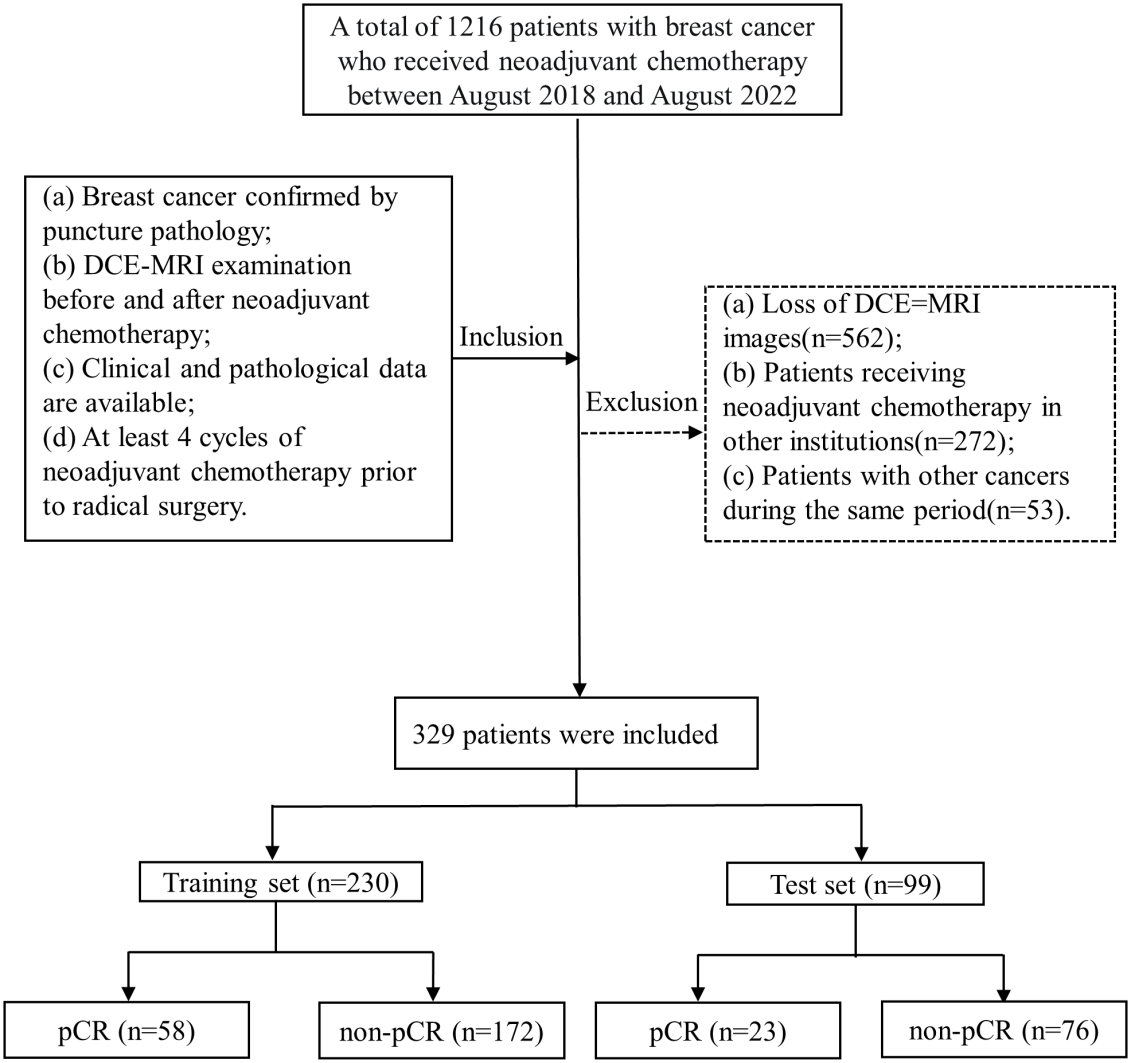
Flow chart of patient enrollment. (MRI, enhanced magnetic resonance imaging).

Patient screening is shown in **Figure 2**. The clinical characteristics of the entire training and test sets are shown in **Table 1**. All characteristics were not significantly different between the training and test sets (p> 0.05). Among all 329 patients, a total of 81 patients achieved pCR, and the pCR probability was 24.6%. The pCR rate was 23.2% in the training set and 25.2% in the test set. This is not considerably different from the pCR rate in previous studies (20, 21). The clinical information between patients in the pCR and non-pCR groups is shown in **Supplementary Table 2**.

**Table 1:** Consistency of clinical characteristics between training set and test set.

Characteristic	No. (%)	Test set(n=99)	p Value
	Training set(n=230)		
Age, years			0.06
≥40	36 (15.7)	25 (25.2)	
<40	195 (84.3)	74 (74.8)	
Menopausal			0.13
Premenopausal	116 (50.4)	59 (59.6)	
Postmenopausal	114 (49.6)	40 (40.4)	
ER			0.32
Negative	90 (39.1)	33 (33.3)	
Positive	140(49.6)	66 (66.7)	
PR			0.15
Negative	113 (49.1)	40 (40.4)	
Positive	117 (50.9)	59 (59.6)	
Her-2			0.38
Negative	132 (57.4)	62 (62.7)	
Positive	98 (42.6)	37 (37.2)	
Ki-67			0.68
≤30%	101 (43.9)	41 (41.4)	
>30%	129 (56.2)	58 (58.6)	
Clinical T stage			0.26
1	24 (10.4)	2 (2.0)	
2	102 (44.3)	54 (54.6)	
3	85 (37.0)	31 (31.3)	
4	19 (8.3)	12 (12.1)	
Clinical N stage			0.78
1	202 (87.8)	88 (88.9)	
2	28 (12.2)	11 (11.1)	

### 3.2 Radiomic model

**Figure 3:**
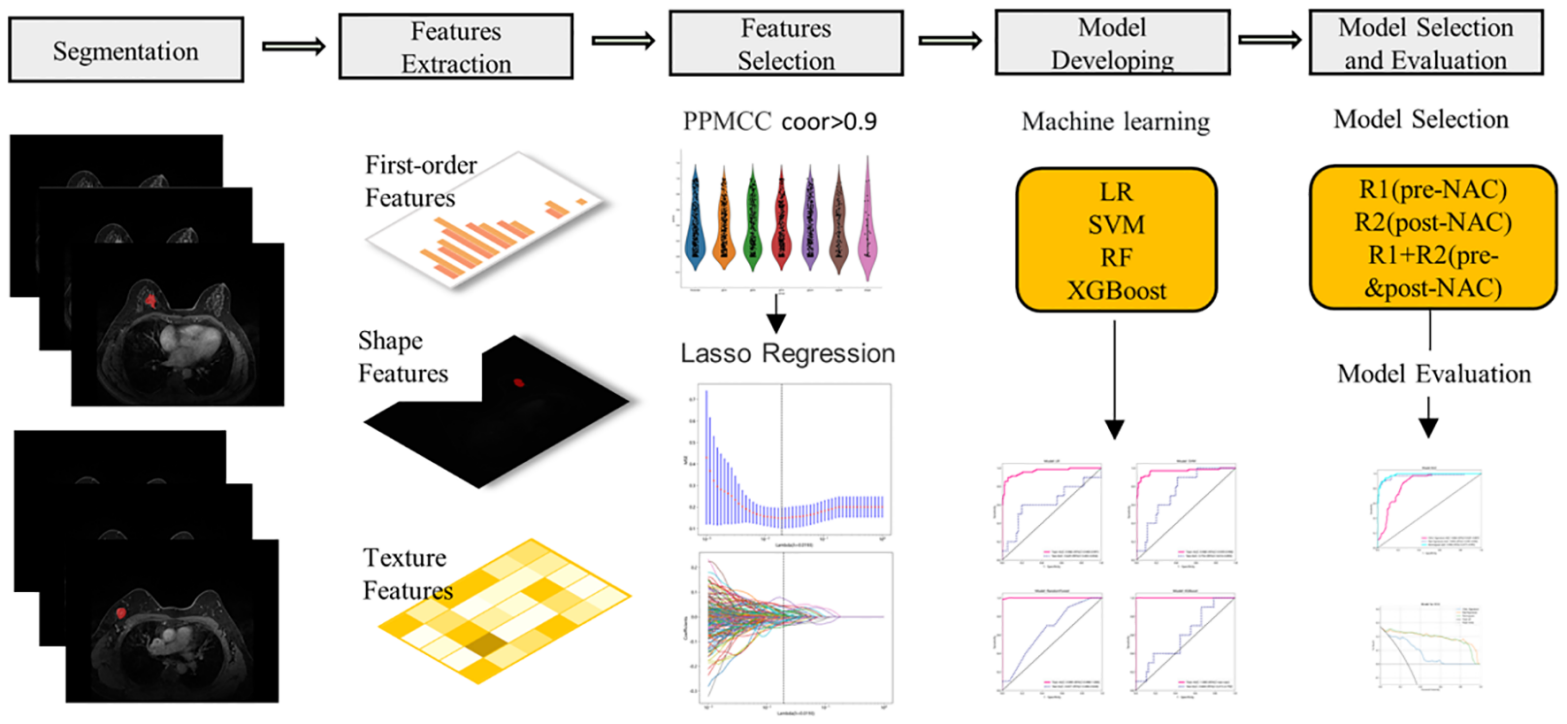
The workflow of MRI radiomics model development. PPMCC, Pearson product-moment correlation coefficient; LASSO, least absolute shrinkage and selection operator; SVM, support vector machine; LR, logistic regression; RF, random forest; XGBoost, eXtreme Gradient Boosting.

**Figure 4:**
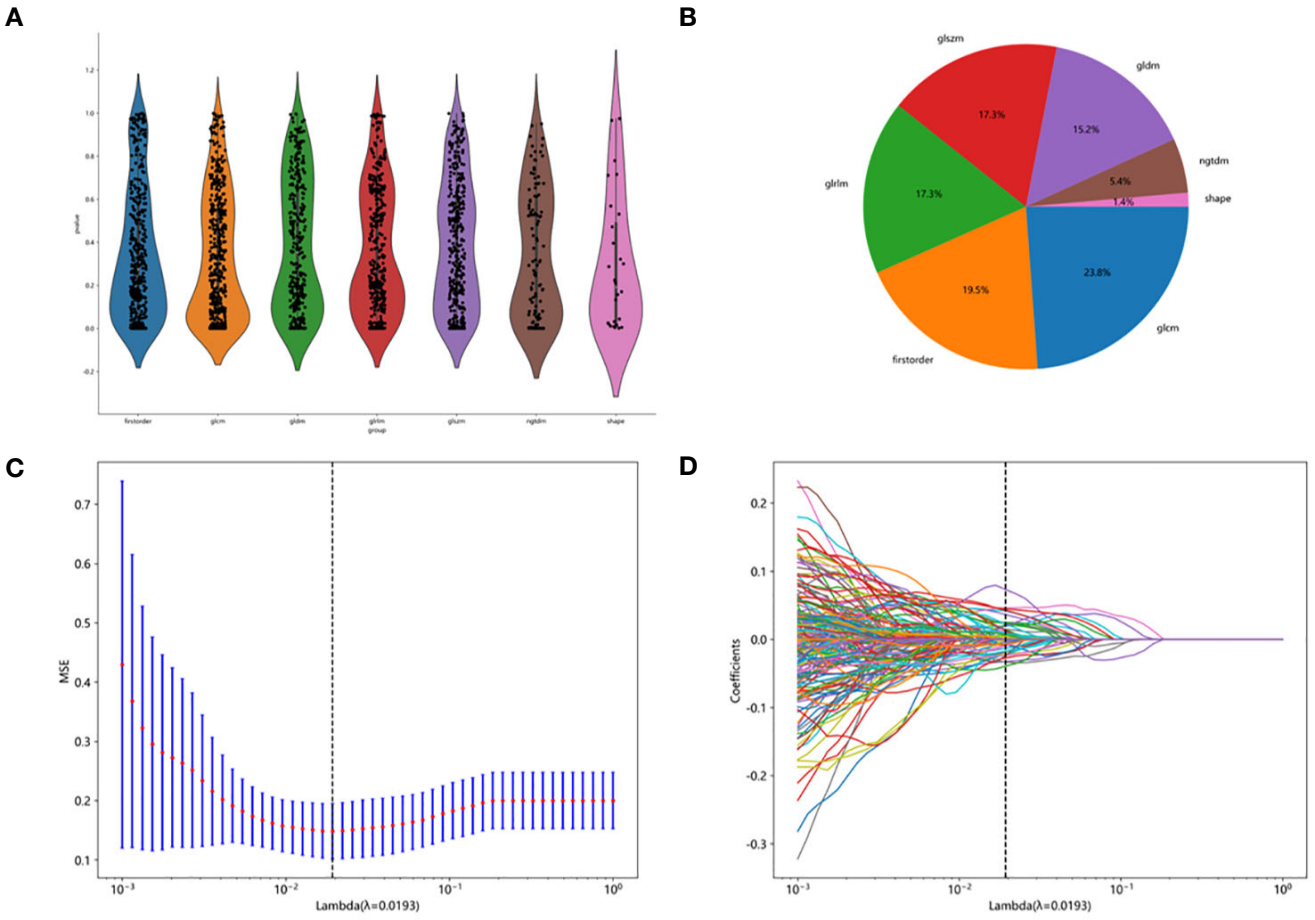
Feature screening procedure. **(A)** distribution map of screening features. **(B)** the proportion of each filter feature. **(C, D)** lasoo regression diagram.

The entire radiomic modeling process is shown in **Figure 3**. We manually segmented MRI images of 329 patients on each layer and then extracted features. A total of 1032256 features were extracted from R1+R2 images, and according to one-way analysis of variance, 4788 meaningful features were screened out, and the Pearson correlation coefficient was determined. If the correlation was greater than 0.9, one of the features was retained. Then, 48 features were further screened by lasso regression (**Figures 4A–D**). We did the same thing in solitary R1 and R2. Four machine learning methods were used for modeling. Based on the machine learning results, we select the best algorithm. In the R1 training set, the AUCs of LR, SVM, RF and XGBoost were 0.765, 0.930, 0.996 and 1.000, respectively. In the R1 test set, the AUC values were 0.730, 0.748, 0.605 and 0.793, respectively. In R2, the AUCs of the four machine algorithms were 0.956, 0.954, 0.998 and 1.000 in the training set and 0.666, 0.761, 0.600, and 0.634 in the test set. The AUCs of R1+R2 were 0.966, 0.968, 0.999, and 1.000 in the training set and 0.629, 0.754, 0.657, and 0.604 in the test set, respectively, which were better than those of R1 and R2 alone. Therefore, we chose the R1+R2 model as the selected radiomic model for comparison with the subsequent model (**Table 2**). Due to its better AUC, sensitivity and specificity of, SVM was used for subsequent comparison.

**Table 2:** Comparison of AUCs in the radiomics model.

Model		LR	AUC(95%CI)		XGBoost
			SVM	RF	
R1	Training	0.765 (0.701-0.830)	0.930 (0.885-0.974)	0.996 (0.990-1.000)	1.000 (1.000-1.000)
	Test	0.730 (0.577-0.884)	0.748 (0.603-0.893)	0.605 (0.399-0.812)	0.793 (0.676-0.910)
R2	Training	0.956 (0.934-0.979)	0.954 (0.923-0.986)	0.998 (0.995-1.000)	1.000 (1.000-1.000)
	Test	0.666 (0.451-0.882)	0.761 (0.604-0.918)	0.600 (0.338-0.812)	0.634 (0.429-0.841)
R1+R2	Training	0.966 (0.940-0.991)	0.968 (0.939-0.996)	0.999 (0.998-1.000)	1.000 (1.000-1.000)
	Test	0.629 (0.609-0.903)	0.754 (0.614-0.828)	0.657 (0.486-0.929)	0.604 (0.415-0.792)

R1: pre-NAC, R2: post-NAC, R3: pre- and post-NAC.

### 3.3 Clinical model

The clinical model yielded AUC values of LR, SVM, RF and XGB of 0.852, 0.864, 0.999, and 0.972 in the training set and 0.823, 0.724, 0.721, and 0.799 in the test set for predicting a pCR (**Supplementary Table 1**). The AUCs of the four models were higher. Due to its better AUC, sensitivity and specificity, SVM was used for subsequent comparison.

### 3.4 Combination model

**Figure 5:**
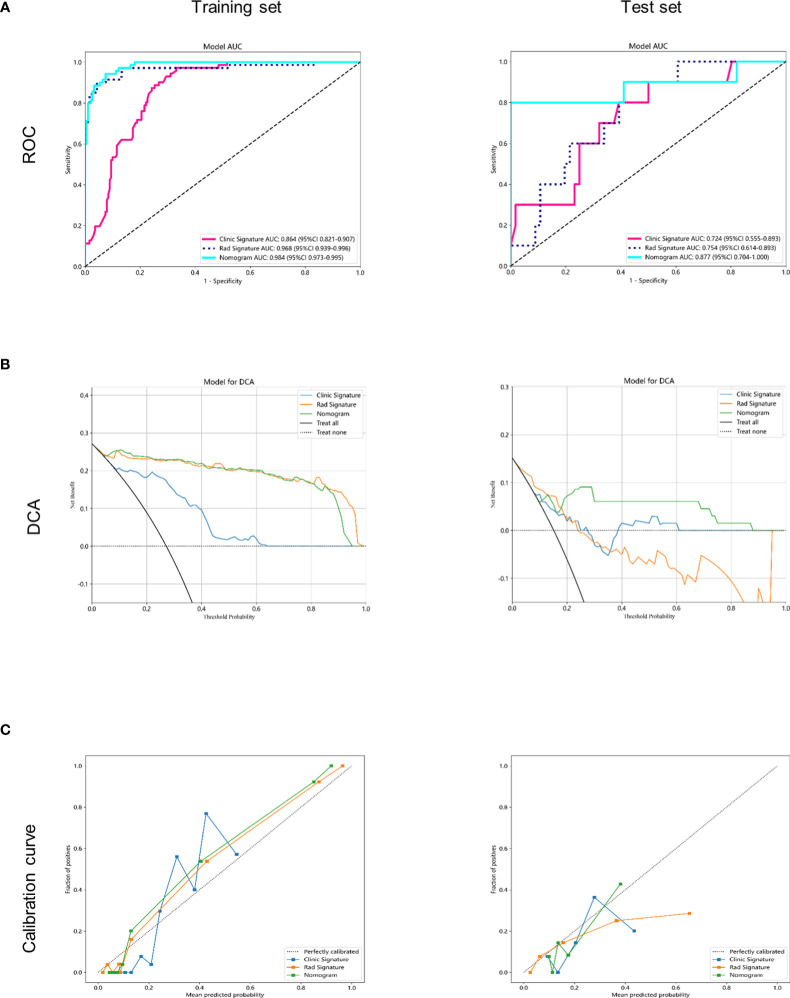
Receiver operating characteristic (ROC) curves and decision curve analysis of the 3 models in the training and testing sets. **(A)** receiver operating characteristic (ROC) curves in the training and testing sets. **(B)** decision curve analysis (DCA) curves in the training and testing sets. **(C)** calibration curves in the training and testing sets.

**Figure 6:**
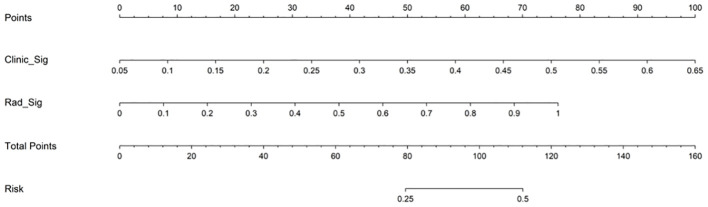
Nomogram to predict pCR after neoadjuvant chemotherapy in breast cancer patients. Different values for each variable correspond to a point at the top of the graph, and the sum of points for all variables corresponds to the total point. The line from the total point to the bottom is the probability of pCR. pCR, pathological complete response; Clinic_Sig, clinical signature; Rad_Sig, radiomics signature.

The AUC of the clinical combined radiomics model in the training set was 0.984 and that in the test set was 0.877 (**Figure 5A**). Combining DCA, we can see that the clinical-radiomic model showed good predictive performance in both the training set and the test set, and it was better than both the clinical model and the radiomic model (**Figure 5B**). We have developed a clinical-radiomic nomogram that is more concise in the end (**Figure 6**). The calibration curve showed good agreement between the probabilities predicted by the nomogram and those observed in the two sets (**Figure 5C**).

## 4 Discussion

The purpose of this study was to establish a model to evaluate whether breast cancer patients will achieve a pathological complete response after NAC by using MRI image analysis and clinical factors. The AUC of our final combined model is 0.984 in the training set and 0.877 in the test set, which is a relatively good result. Prior to traditional radiomics, studies have used imaging features to predict the efficacy of neoadjuvant chemotherapy, with promising results. For example, the La Forgia D et al. (22)study used background parenchymal enhancement (BPE) parameters in breast magnetic resonance imaging (MRI) as potential predictors of neoadjuvant therapy. With the development of imagomics, more precise feature extraction processes allow us to make more accurate predictions. The advantages of this study are the use of relatively many samples and a relatively standardized radiomics extraction process, making the process more comparable and reproducible.

Based on previous studies, eight clinical and pathological factors, including age, clinical T stage and N stage, menopausal state, ER, PR, Her-2 and Ki-67, were selected to predict pCR. Pierga et al. (23) demonstrated that pathological lymph node status after NAC was a major prognostic factor for patients. Verdial’ et al. (24)study showed that young women (≤ 40 years of age) with breast cancer who received neoadjuvant chemotherapy (NAC) had a higher incidence of pathological complete response (pCR). A meta-analysis showed that patients with a high Ki-67 marker index showed significantly higher pCR rates. Mernut et al. (25) found that T1 staging, N1 staging, NG 3, estrogen receptor negative, progesterone receptor negative, Her-2 positive, and chemotherapy plus trastuzumab for patients with pCR were statistically significant (p<0.05). Amoroso N et al. ‘s results show that molecular subtypes are the most important features of hierarchical clustering (26). Considering that the number of selected predictors after univariate analysis and multifactor analysis is small, unable to reflect most clinical information of patients, and has little clinical adaptability, we intended to incorporate all eight clinical factors into the model construction. Tsai et al. (27) established a model based on the radiomic features and clinical features of CT, and all 6 clinical features were used in the construction of the clinical model. The final AUC was 0.69, showing moderate performance, while the AUCs of our clinical model were 0.864 and 0.781, showing better efficacy. The best predictive efficacy was achieved when the machine learning model included both clinical and radio-MRI parameters, which is instructive for subsequent treatment of patients. This has also been shown in studies of other tumors, such as locally advanced cervical cancer (28).

To achieve robust and high performance of the classifiers, four machine learning algorithms, LR, SVM, RF, and XGBoost, were used for classifier construction. In this study, these algorithms were selected based on the common classifiers used in previous studies on mammary glands, such as breast cancer prediction, axillary lymph node metastasis, and mastectomy (29–31). To avoid overfitting in the modeling process, we used the grid search method and tenfold cross validation to repeatedly complete the hyperparameter search of the optimal classifier. In the R1+R2 training set, the AUC values of four machine learning classifiers ranged from 0.900 to 1.000, and SVM classifiers showed the best performance, while the others were all above 0.9. The DeLong test found no significant difference. In the test set, SVM classifiers performed best, with AUCs ranging from 0.6 to 0.8. The sensitivity and specificity of SVM were 0.9 and 0.957 in the training set and 0.9 and 0.589 in the test set. Therefore, SVM classifiers were finally selected and obtained as the Rad-Score model. In the process of radiomics training, the loss of backpropagation is obtained through the training set, while the test set does not participate in the training process, so it is normal that the accuracy of the training set is slightly higher than that of the test set. On the contrary, if the accuracy of the training set cannot be guaranteed, the accuracy of the test set will generally be worse. However, our training accuracy is 0.968, and the test accuracy is 0.754. The difference is not large, there is only a slight overfitting that does not affect the overall effect, which is a normal phenomenon in the training process, and can be further alleviated by increasing the amount of data in the future. A comparison of the ROC curves of the four machine learning classifiers in the training set and test set is shown in **Table 2**.

Due to the many types of MRI radiomic features, the specific radiomic features ultimately selected for modeling are mostly different according to different research purposes and methods. For example, Li’ et al. (32) established multiparameter MRI radiomic models of tumors and their surroundings. A total of 863 radiomic features were extracted, and the first 30 features were selected for the final model construction by the RF algorithm. These features were selected based on correlation. Sheng et al. (33) combined dynamic contrast-enhanced magnetic resonance imaging (DCE-MRI) three-dimensional volume characteristics with clinical data to predict molecular subtypes of invasive ductal breast cancer using t tests and LASSO regression in R language for screening. The final features included shape-based features, texture features, wavelet features, first-order statistical features and the Laplacian of a Gaussian filter. The features extracted by the best radiomics model in this study included shape features, signal intensity features, texture features and high-order features, among which shape features were very important in the evaluation of tumor features (4). By extracting features from lymph nodes and primary tumors separately and analyzing them together, we can more convincingly include shape features. Texture features explained the spatial interdependence or cooccurrence of information between adjacent voxels, which could be used to assess intertumor heterogeneity and reflect lymphocyte infiltration and molecular subtypes of breast tumors (34).

Most of the studies in the past literature involved establishing separate radiomic models, such as separate pre-NAC. Choudhery et al. (35) retrospectively analyzed clinical and pretreatment MRI data of 259 patients with biopsy-confirmed breast cancer receiving NAC and found that the radiomic features were associated with different molecular subtypes, pCR and RCB. Liu et al. (36) extracted 10 quantitative imaging features from T2-weighted, diffusion-weighted, and enhanced T1-weighted imaging before NAC in each patient and developed a new RMM model based on optimal radiological features combined with independent clinicopathological risk factors to predict pCR for NAC in breast cancer patients. The RMM model was better than the clinical model and the radiomics model. We suggest that combining the radiomic features of MRI after NAC can reduce the influence of confounding factors such as different treatment regiments and cycles on predicting pCR. Furthermore, previous studies have focused on the use of intertumor and peritumor features without considering the association between ALNs and the primary tumor. Trials such as Braman et al. (18) evaluated intratumor and peritumor radiomic features in 117 patients, with a maximum AUC of 0.78 ± 0.03 for the combined feature set. Our results also showed that the R1+R2 model was superior to either R1 or R2. Therefore, our study not only accounted for pretreatment and posttreatment image features but also combined axillary lymph nodes with the primary tumor, including as many radiomic features as possible, to achieve a better prediction effect. According to the CSCO guidelines, neoadjuvant chemotherapy is recommended for the following patients: (1) large masses (> 5cm); (2) axillary lymph node metastasis; (3) Positive HER-2; (4) Triple negative; (5) There is a desire to preserve the breast, but the ratio of tumor size to breast volume is large and difficult to preserve the breast. Our center started neoadjuvant therapy early, but the patients who received neoadjuvant therapy were mostly patients with N+ and T3-4. Woo et al. (37) retrospectively analyzed 1017 patients who received NAC and surgery, and found that 16.2% of patients obtained a breast radiologic complete remission (rCR) and 26.9% obtained an axillary rCR. The proportion of this population was not high. The patients we included this time were all N+, so the delineation of breast masses was mainly based on the fibrotic changes after tumor regression, and the axillary lymph nodes were based on the anatomical location and chemotherapy changes. The ROI were sketched by senior doctors after drawing by junior doctors, and the disputed parts were revised jointly by senior doctors, so as to increase accuracy.

However, our study still has many limitations. First, all of our patients were from the same institution, we enrolled a limited number of patients, we did not use a multicenter collaborative study, and we conducted only internal validation, not external validation. Secondly, this is a retrospective study, and future prospective studies are needed to verify our column graph. Third, when extracting the radiomic features, only T1 was selected, and T2 and DWI phases were not included. Moreover, manual segmentation was chosen when we were making the ROI, which was relatively time-consuming and labor-intensive. Semiautomatic or automatic mapping could be considered to help reduce the interobserver radiomic feature variability caused by manual segmentation in the included study. Then, the patients we included were all N+, it is also hoped to explore the pCR prediction of non-N + patients in the future. The last, there was no classification analysis for breast cancer patients; otherwise, predicting different types or distinguishing different neoadjuvant chemotherapy schemes to provide personalized treatment might have been possible.

## 5 Conclusions

In summary, we showed that the combination of clinical factors and radiological characteristics could be used to better predict whether breast cancer patients achieve pCR after neoadjuvant chemotherapy and proposed a nomogram model. The model was helpful in treatment decision-making for the individualized treatment of breast cancer patients, but more patients or prospective experiments are still needed to verify the conclusions in the future.

## Data availability statement

The original contributions presented in the study are included in the article/**Supplementary Material**, further inquiries can be directed to the corresponding author/s.

## Ethics statement

This single-center, retrospective study was approved by our Institutional Review Board, and the requirement for written informed consent was waived.

## Author contributions

YY designed the study and wrote the manuscript; ZW analyzed the data; QW designed the study; XS, ZL, and RW collected data; TG, WG, and HW reducted data; BZ supervised and writed final draft. All authors contributed to the article and approved the submitted version.
