# Supplementary material for: Radiomic model based on magnetic resonance imaging for predicting pathological complete response after neoadjuvant chemotherapy in breast cancer patients
Source: Front Oncol. 2024 Jan 31;13:1249339. doi: 10.3389/fonc.2023.1249339 (PMC10865896; doi:10.3389/fonc.2023.1249339)
Supplement: Supplementary file 1 [file Table_1.docx]

**Supplementary Table 1.** Comparison of AUCs in the clinical model

| Model | AUC (95%CI) | | | |
| --- | --- | --- | --- | --- |
|  | LR | SVM | RF | XGBoost |
| Clinical |  |  |  |  |
| Training | 0.852 (0.806-0.897) | 0.864 (0.819-0.911) | 0.999 (0.996-1.000) | 0.972 (0.955-0.989) |
| Test | 0.823 (0.711-0.934) | 0.724 (0.555-0.893) | 0.721 (0.575-0.868) | 0.799 (0.679-0.915) |

Notes: LR, logistic regression; SVM, support vector machine; RF, random forest; XGBoost, eXtreme Gradient Boosting.
