# Supplementary material for: Radiomic model based on magnetic resonance imaging for predicting pathological complete response after neoadjuvant chemotherapy in breast cancer patients
Source: Front Oncol. 2024 Jan 31;13:1249339. doi: 10.3389/fonc.2023.1249339 (PMC10865896; doi:10.3389/fonc.2023.1249339)
Supplement: Supplementary file 2 [file Table_2.docx]

| **Supplementary Table 2.** Clinical characteristics between patients with pCR and nonpCR | | | | |
| --- | --- | --- | --- | --- |
| **Characteristic** | **No. (%)** | |  | **p Value** |
|  | **pCR（n=81）** | **nonpCR（n=248）** | |  |
| Age，years |  |  | | 0.97 |
| ≥40 | 64 (79.0) | 205 (82.7) | |  |
| ＜40 | 17 (21.0) | 43 (17.3) | |  |
| Menopausal |  |  | | 0.24 |
| Premenopausal | 42 (51.9) | 133 (53.6) | |  |
| Postmenopausal | 39 (48.1) | 115 (46.4) | |  |
| ER |  |  | | 0.09 |
| Negative | 54 (66.7) | 69 (38.5) | |  |
| Positive | 27(33.3) | 179 (66.7) | |  |
| PR |  |  | | 0.28 |
| Negative | 59 (72.8) | 94 (37.9) | |  |
| Positive | 22 (27.2) | 154 (62.1) | |  |
| Her-2 |  |  | | ＜0.01 |
| Negative | 16 (19.8) | 178 (71.8) | |  |
| Positive | 65 (80.2) | 70 (28.2) | |  |
| Ki-67 |  |  | | 0.08 |
| ≤30% | 24 (29.6) | 118 (47.6) | |  |
| ＞30% | 57 (70.4) | 130 (52.4) | |  |
| Clinical T stage |  |  | | 0.14 |
| 1 | 10 (12.3) | 16 (6.5) | |  |
| 2 | 25 (30.9) | 121 (48.8) | |  |
| 3 | 35 (43.2) | 91 (36.7) | |  |
| 4 | 11 (13.6) | 20 (8.0) | |  |
| Clinical N stage |  |  | | 0.317 |
| 1 | 73 (90.1) | 217 (87.5) | |  |
| 2 | 8 (9.9) | 31 (12.5) | |  |
